# Meningococcal Factor H Binding Proteins in Epidemic Strains from Africa: Implications for Vaccine Development

**DOI:** 10.1371/journal.pntd.0001302

**Published:** 2011-09-06

**Authors:** Rolando Pajon, Andrew M. Fergus, Oliver Koeberling, Dominique A. Caugant, Dan M. Granoff

**Affiliations:** 1 Center for Immunobiology and Vaccine Development, Children's Hospital Oakland Research Institute, Oakland, California, United States of America; 2 Department of Bacteriology and Immunology, Norwegian Institute of Public Health, and Department of Community Medicine, University of Oslo, Oslo, Norway; Mahidol University, Thailand

## Abstract

**Background:**

Factor H binding protein (fHbp) is an important antigen for vaccines against meningococcal serogroup B disease. The protein binds human factor H (fH), which enables the bacteria to resist serum bactericidal activity. Little is known about the vaccine-potential of fHbp for control of meningococcal epidemics in Africa, which typically are caused by non-group B strains.

**Methodology/Principal Findings:**

We investigated genes encoding fHbp in 106 serogroup A, W-135 and X case isolates from 17 African countries. We determined complement-mediated bactericidal activity of antisera from mice immunized with recombinant fHbp vaccines, or a prototype native outer membrane vesicle (NOMV) vaccine from a serogroup B mutant strain with over-expressed fHbp. Eighty-six of the isolates (81%) had one of four prevalent fHbp sequence variants, ID 4/5 (serogroup A isolates), 9 (W-135), or 74 (X) in variant group 1, or ID 22/23 (W-135) in variant group 2. More than one-third of serogroup A isolates and two-thirds of W-135 isolates tested had low fHbp expression while all X isolates tested had intermediate or high expression. Antisera to the recombinant fHbp vaccines were generally bactericidal only against isolates with fHbp sequence variants that closely matched the respective vaccine ID. Low fHbp expression also contributed to resistance to anti-fHbp bactericidal activity. In contrast to the recombinant vaccines, the NOMV fHbp ID 1 vaccine elicited broad anti-fHbp bactericidal activity, and the antibodies had greater ability to inhibit binding of fH to fHbp than antibodies elicited by the control recombinant fHbp ID 1 vaccine.

**Conclusion/Significance:**

NOMV vaccines from mutants with increased fHbp expression elicit an antibody repertoire with greater bactericidal activity than recombinant fHbp vaccines. NOMV vaccines are promising for prevention of meningococcal disease in Africa and could be used to supplement coverage conferred by a serogroup A polysaccharide-protein conjugate vaccine recently introduced in some sub-Saharan countries.

## Author Summary

Epidemics of meningococcal meningitis are common in sub-Saharan Africa. Most are caused by encapsulated serogroup A strains, which rarely cause disease in industrialized countries. A serogroup A polysaccharide protein conjugate vaccine recently was introduced in some countries in sub-Saharan Africa. The antibodies induced, however, may allow replacement of serogroup A strains with serogroup W-135 or X strains, which also cause epidemics in this region. Protein antigens, such as factor H binding protein (fHbp), are promising for prevention of meningococcal serogroup B disease. These proteins also are present in strains with other capsular serogroups. Here we report investigation of the potential of fHbp vaccines for prevention of disease caused by serogroup A, W-135 and X strains from Africa. Four fHbp amino acid sequence variants accounted for 81% of the 106 African isolates studied. While there was little cross-protective activity by antibodies elicited in mice by recombinant fHbp vaccines from each of the four sequence variants, a prototype native outer membrane vesicle (NOMV) vaccine from a mutant with over-expressed fHbp elicited antibodies with broad protective activity. A NOMV vaccine has the potential to supplement coverage by the group A conjugate vaccine and help prevent emergence of disease caused by non-serogroup A strains.

## Introduction

For more than 100 years devastating epidemics of meningococcal disease have occurred in sub-Saharan Africa [1]. In the decade 1988 to 1997, more than 700,000 cases and over 100,000 deaths were reported. Public health responses were limited by scarce resources [2]. Further, the only vaccines available, un-conjugated (plain) polysaccharides, elicited incomplete and short duration of protection in young children [3], [4], and had a minimal effect on decreasing transmission of the organism [3], [5], [6]. Control of epidemic meningococcal disease in Africa, therefore, remains an unsolved public health challenge.

Most meningococcal disease in industrialized countries is caused by strains producing capsular serogroups B, C or Y, whereas most disease in sub-Saharan Africa is caused by serogroup A strains. After more than ten years of work [7], [8], a promising serogroup A polysaccharide-protein conjugate vaccine recently was developed for sub-Saharan Africa [9], [10]. As of January 21, 2011, nearly 20 million people had been immunized as part of demonstration projects in three countries (http://www.path.org/menafrivac/index.php). While this vaccine has the potential to eliminate serogroup A epidemics, widespread vaccination may result in selective pressure for replacement of strains with other capsular serogroups such as X or W-135, which have caused epidemics in this region [11]–[14]. However, with the possible exception of Spain [15], there is little evidence of serogroup replacement after widespread use of monovalent serogroup C meningococcal conjugate vaccines in Europe [16], [17]. Pneumococcal serotype replacement, in contrast, has been a problem in many countries where pneumococcal polysaccharide-protein conjugate vaccines were introduced [18]–[22].

A number of protein antigens are being developed for prevention of meningococcal serogroup B disease (Reviewed in [23], [24]). These antigens also are prevalent in meningococcal strains with other capsular serogroups [7], [25], [26]. Therefore, the vaccines have the potential to prevent disease caused by non-group B strains. One of the most promising of the new protein vaccine candidates is factor H binding protein (fHbp, which was previously referred to as GNA1870 [27] or LP2086 [28]). Recombinant fHbp is part of two vaccines in clinical development for prevention of serogroup B disease [28]–[30]. Native outer membrane vesicle vaccines from meningococcal mutants with over-expressed fHbp also are under investigation [31]–[36]. The fHbp antigen is a surface-exposed lipoprotein that binds complement fH [37], which down-regulates complement activation and enhances the ability of the organism to escape complement-mediated bacteriolysis [37]–[39]. In immunized mice and humans, antibodies to recombinant fHbp vaccines elicited complement-mediated serum bactericidal activity [27], [28], [30], [40]–[45], which in humans is the hallmark of protection against meningococcal disease [46]–[48]. The present investigation was undertaken to determine the vaccine-potential of fHbp for control of meningococcal epidemics in Africa caused by serogroup A, W-135 and X isolates.

## Materials and Methods

### Objectives

In a previous study, we characterized fHbp sequence variants in a small collection of serogroup A, W-135 and X isolates from patients in sub-Saharan Africa [49]. The objectives of the present study were to determine fHbp sequence variants in an expanded panel of case isolates from Africa, to measure levels of fHbp expression, which in previous studies had been reported to be important for predicting susceptibility of serogroup B strains to anti-fHbp bactericidal activity [50], and to investigate the immunogenicity in mice of recombinant fHbp vaccines representative of sequence variants prevalent among invasive African strains. The recombinant vaccine immunogenicity results were compared to that of a prototype native outer membrane vesicle vaccine (NOMV) prepared from a serogroup B mutant strain engineered to over-express fHbp. Our hypothesis was that the NOMV would elicit broader serum bactericidal antibody responses against the strains from Africa than the recombinant fHbp vaccines since in previous studies, mutant NOMV vaccines with over-expressed fHbp elicited broader bactericidal activity against serogroup B strains [33]–[36], [51].

### Descriptions of procedures

#### Isolates

**Figure 1:**
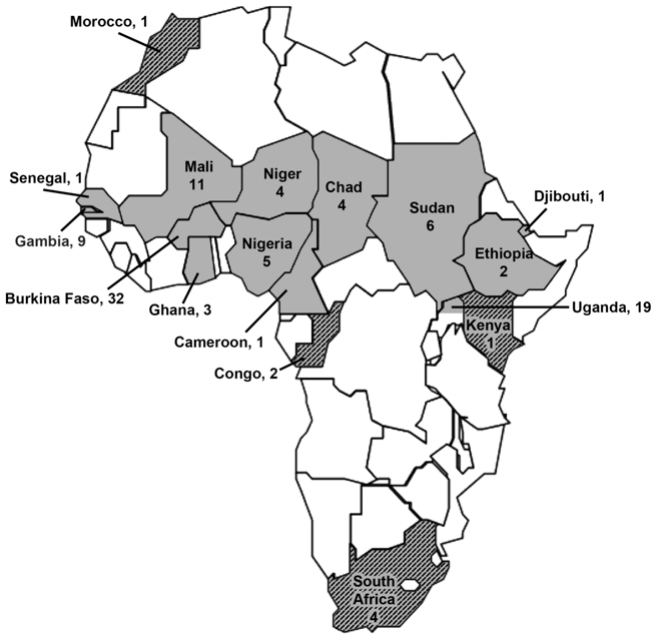
Map of Africa showing the source countries (gray) for the isolates and the number of isolates from that country. Countries shown in gray with hatches are from outside the meningitis belt. Gulu district in northern Uganda is part of the meningitis belt (see text).

The 106 isolates were from 17 countries (Figure 1). Ninety-eight of the isolates were from patients residing within the meningitis belt, which included the northern district of Gulu, Uganda (http://www.who.int/csr/don/2006_03_21/en/index.html)), and eight isolates were from outside the belt (Figure 1, four countries in gray with hatches). These eight isolates included three serogroup X and two serogroup A from 1967 to 1989, and one serogroup A, X, and W-135 isolate from 2001 to 2006. The 106 isolates were selected from a collection of more than 600 case isolates from Africa that date back to the early 1970s, at the WHO Collaborating Centre for Reference and Research on Meningococci, Norwegian Institute of Public Health. Because individual epidemics are usually clonal, selection of isolates for the present study was limited to no more than 10 isolates from any one epidemic in a restricted geographic area and year. We also included only serogroup A (N = 31), W-135 (N = 53) or X (N = 22) isolates since strains with these capsular serogroups were responsible for nearly all meningococcal epidemics in Africa in recent decades. Since a promising serogroup A conjugate vaccine was under development for Africa, we included a disproportionate number of serogroup W-135 and X isolates to provide data on the vaccine-potential of fHbp to extend coverage to non-group A strains. The 32 isolates from Burkina Faso were from a 45-year period, and included 22 serogroup W-135 isolates between 2001 and 2004, six serogroup A isolates from 1963, 1966, 2003 and 2007, and 4 serogroup X isolates from 1997, 2003, and 2007. For all 106 isolates, the year of isolation, country of origin, capsular serogroup, multilocus sequence type, fHbp sequence variant, and PorA variable region (VR) type are provided in Table S1.

To investigate the effect of fHbp expression on susceptibility of an isolate to anti-fHbp bactericidal activity we engineered isogenic mutants from serogroup A, B, and W-135 isolates to have different levels of fHbp expression. The serogroup A isolate, A3 (also designated Senegal 1/99), was a naturally low fHbp expresser. The serogroup W-135 isolate, W13 (also designated Sudan 1/06), was a naturally intermediate fHbp expresser, while the serogroup B isolate, H44/76, was a naturally high fHbp expresser (See results). Generation of the respective engineered isolates was made through modifications of promoters previously described [51].

#### Multilocus sequence and *porA* typing

Isolates were assigned to a specific sequence type (ST) using multilocus sequence typing (MLST) as described at (http://pubmlst.org/neisseria/). Variation in the *porA* gene was determined by DNA sequencing [52], using an ABI 3730 DNA analyzer (Applied Biosystems).

#### PCR and DNA sequencing of fHbp genes

The fHbp genes were PCR-amplified from heat-killed cell samples of the isolates using A1 and B2 primers previously described [27]. DNA sequences of the PCR products were determined using the A1 and/or 22 primers [27] (Davis Sequencing LLC, Davis, CA). The analysis presented included 84 newly described sequences and fHbp sequences from 22 isolates described in a previous study [49].

Based on analysis of amino acid sequence variants, fHbp antigens have been subdivided into two sub-families [28], three variant groups [27], or six major modular groups [50], [53]. For purposes of vaccine development and serological classification we used the three antigenic variant groups originally described by Masignani et al [27], which designated sub-family B as variant group 1, and sub-divided sub-family A fHbp sequences into variant groups 2 and 3.

#### Cloning, expression and purification of recombinant proteins

Expression plasmids were constructed by PCR amplification of fHbp genes, which was performed as described previously [50]. Genes encoding four prevalent fHbp sequence variants, ID 4, 9 and 74 (variant group 1), and ID 22 (variant group 2), were cloned into pET21b. C-terminal hexahistidine-tagged recombinant fHbps were expressed in *Escherichia coli* BL21(DE3) (Novagen, Madison, WI, US), and purified as described elsewhere [40]. Additional control recombinant fHbp vaccines were prepared using similar methods from fHbp genes encoding ID 1 (variant 1 group), 77 (variant 2 group) and 28 (variant 3 group).

#### Mouse antisera

Groups of five-week-old CD-1 female mice (10 mice per group) were obtained from Charles River (Wilmington, MD, US). For generation of hyperimmune sera, the mice were immunized intra-peritoneally (IP) with three doses of recombinant fHbp ID 4, 9, 74, or 22 vaccines, or the control fHbp ID 1, 77 or 28 vaccines. Each 100 µl dose contained 20 µg of recombinant protein mixed with Freund's complete adjuvant (FA) (Sigma-Aldrich, St. Louis, MO, US) for the first dose and Freund's incomplete adjuvant for doses 2 and 3, which were given at 2-week intervals. Terminal blood samples were obtained three weeks after the last dose. The sera from the mice immunized with the respective fHbp ID vaccines were pooled.

We also investigated the vaccine-potential of a prototype NOMV vaccine prepared from a mutant of group B strain H44/76 that had been engineered to over-express fHbp ID 1, as previously described [36], [54]. The phenotype of the parent wildtype strain was B:15:P1.7,16; fHbp ID 1, ST-32, and LOS L 3,7,9. Control vaccines consisted of a NOMV vaccine prepared from a H44/76 mutant in which the gene encoding fHbp had been inactivated [54], or recombinant fHbp ID 1.

The NOMV vaccines consisted of native outer membrane blebs that were spontaneously released into the culture supernatant during growth of the bacteria. In brief, bacteria were grown to early stationary phase (OD 1.0 to 1.2 after 6 hours of growth); phenol 0.5% (w/v) was added. After overnight incubation at 4°C to inactivate the bacteria, the culture was centrifuged for 10 minutes at 7500 x g to pellet the bacteria. NOMVs were harvested from the culture supernatant by the addition of 390 g/l of ammonium sulfate. The mixture was left overnight at 4°C and the precipitate was collected by centrifugation at 7500 x g. The precipitate was suspended in 40 ml PBS, and the NOMV in the supernatant was collected by centrifugation at 100,000 x g for 2 hours. The isolated blebs were re-suspended in 3% sucrose, and stored at – 20°C before use.

Based on Western blot, fHbp was detected in the NOMV vaccine from the mutant with over-expressed fHbp but not in the fHbp knockout mutant (Figure S1, Panel A). By Coomassie-stained SDS-PAGE, the two NOMV vaccines contained similar levels of the major outer membrane proteins PorA and PorB (Panel B); the NOMV from the mutant with over-expressed fHbp showed higher amounts of a band resolving at ∼30kD, which in a previously published study was demonstrated by mass spectrometry to contain both fHbp and OpcA [34]. Based on silver-stained SDS-PAGE, the respective lipooligosaccharide profiles of the two NOMV vaccines were similar (Panel C).

For immunization with the NOMV vaccines, each CD1 mouse received 1.25 µg of total protein, which was adsorbed with 170 µg of aluminum hydroxide (2% Alhydrogel). Three injections were given, each separated by 3 weeks and terminal blood samples were obtained three weeks after the last dose.

#### Anti-fHbp antibody ELISA

Serum anti-fHbp antibody responses were measured to recombinant wild-type fHbp ID 1 as previously described [55]. The concentrations of antibody in the sera from individual mice were assigned in comparison with binding of anti-fHbp antibody in a reference serum pool from mice immunized with the recombinant fHbp ID 1 vaccine. The reference pool was assigned an arbitrary antibody concentration of 1000 units per ml (U/ml).

#### Anti-fHbp antibody inhibition of binding of fH to fHbp

We tested the ability of serum anti-fHbp antibodies to inhibit binding of human fH to fHbp by ELISA, which was performed as previously described [55] with the following modifications. The antigen on the plate was recombinant fHbp ID 4, which was heterologous to the fHbp ID 1 present in both the recombinant fHbp and fHbp mutant NOMV vaccines (96% amino acid identity with ID 1). We also used 5 µg/ml of purified human fH (Calbiochem, San Diego, CA) in the final reaction mixture instead of IgG-depleted human serum as the source of fH. The percent inhibition by different dilutions of the immune mouse sera was calculated by comparison with the amount of human fH bound in the absence of the mouse antiserum.

#### Complement-dependent serum bactericidal antibody activity

Serum bactericidal titers were measured as previously described using early log-phase bacteria grown for approximately 2 h in Mueller-Hinton broth (BD Biosciences, Franklin Lakes, NJ, US) supplemented with 0.25% glucose (w/v) and 0.02 mM cytidine 5′-monophospho-N-acetylneuraminic acid (Sigma-Aldrich, St, Louis, MO, US) [50]. The complement source was IgG depleted human serum, which was prepared as described [55].

#### fHbp expression

fHbp expression was measured in a subset of isolates from each capsular serogroup. Selection included representative isolates from different countries and different years of isolation that spanned 45 years, and which had genes encoding one of the four predominate fHbp sequence variants, ID 4/5, 9 or 74 (variant group 1), or ID 23 (variant group 2) (See isolates in Table S1 with “secondary isolate designations”). Expression of fHbp was measured by a quantitative Western blot, which had a linear range between 0.1 to 2 µg/ml and was performed as previously reported [50]. For fHbp sequences in variant group 1, anti-fHbp mAb JAR 3 was used for detection of fHbp sequence variants ID 1, 4 or 9, and JAR 5 was used for ID 74 [56]. For fHbp in variant groups 2 or 3, anti-fHbp mAb JAR 31 was used [44]. fHbp expression by the test strains was reported as percentages of the amount of fHbp expressed by bacterial cells from the corresponding reference strains H44/76 or 8047 with high expression of fHbp variant 1 (ID 1) and 2 (ID 77), respectively.

#### Flow cytometry

We measured binding of mouse anti-fHbp mAbs to live meningococcal mutants engineered to have increased or decreased fHbp to assess the relative amounts of fHbp on the bacterial surface. The flow cytometry assay was performed as described previously using a combination of two mouse anti-fHbp mAbs, JAR 4 and JAR 5 [45], each at a final concentration of 10 µg/mL. Controls in the assay included a mouse mAb, specific for serogroup A (JW-A1) or B (SEAM 12) polysaccharides [57].

### Ethics statement

Vaccine immunogenicity was evaluated in CD 1 mice in strict accordance with the recommendations in the Guide for the Care and Use of Laboratory Animals of the National Institutes of Health. The protocol was approved by the Children's Hospital & Research Center at Oakland Institutional Animal Care and Use Committee. Blood collection was performed under anesthesia, and all efforts were made to minimize suffering. The human complement source for measuring serum bactericidal activity was serum from an adult who participated in a protocol that was approved by the Children's Hospital Oakland Institutional Review Board (IRB). Written informed consent was obtained from the subject.

### Statistical analyses

Antibody concentrations were transformed (Log10). For calculations of geometric mean antibody concentrations, concentrations below the limit of the assay were assigned a concentration half of the lower limit. Two-tailed Student's t tests were used to compare the geometric mean antibody concentrations between two independent groups of mice. All statistical tests were two-tailed; probability values of less than or equal to 0.05 were considered statistically significant.

## Results

### The strains from Africa were derived from a limited number of clonal complexes and PorA sequence variants

**Figure 2:**
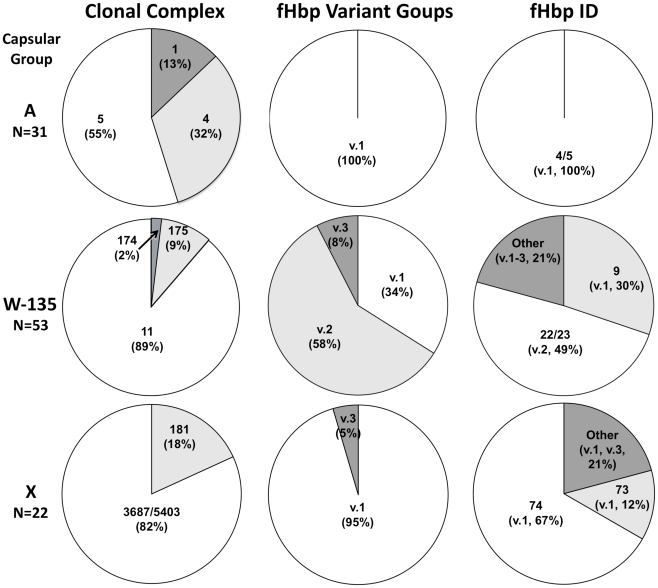
Distribution of clonal complexes (CC), fHbp variant groups, and fHbp sequence variant IDs. CCs were defined by multilocus sequence typing (MLST) with designations described at http://pubmlst.org/neisseria/. For serogroup X isolates, ST 3687 and 5403 differed by a single nucleotide change at one of the seven loci (*fumC).* For purposes of analyses, these isolates were combined as an undesignated 3687/5403 CC. fHbp variant groups 1, 2 or 3, as described by Masignani et al [27], and fHbp amino acid sequence variant (peptide) IDs as provided at http://pubmlst.org/neisseria/. For analyses, respective sequence variants differing by one amino acid were combined (i.e., ID 22/23 includes ID 22 and ID 23).

The distributions of the prevalent clonal complexes, fHbp variant groups, and fHbp sequence variants are shown in Figure 2. The serogroup A isolates were derived from three clonal complexes: CC 1 (three isolates from 1963 to 1967 and one from 1989), CC 4 (ten from 1966 to 1990), and CC 5 (three from 1988–1999, and fourteen from 2003–2007). In contrast, the 53 serogroup W-135 isolates were predominately from CC 11 (N = 47 from 1994 to 2007). Among the 22 serogroup X isolates, four were members of a new CC (designated CC 181 (http://pubmlst.org/neisseria/). Of the remaining 18 isolates, three from the 1970′s were sequence type (ST) 3687, and 15 from 2006–2007 were ST 5403. These two STs differed by only a single nucleotide change in one of the seven loci (*fumC)* and, thus, may represent an undesignated CC, which accounted for 82% of the serogroup X isolates.

There also were a limited number of PorA VR types (Figure S2). Among the serogroup A isolates, P1.20,9 was present overall in 55 percent, and in 89 percent of 18 serogroup A isolates obtained since 1990. Among the serogroup W-135 isolates, P1.5,2 and related types such as 5-1,2-2 predominated (98%), and among the serogroup X isolates, P1.19,26 and related type P1.19,26-4 accounted for 68%. The PorA VR typing results are consistent with previous studies of strains from sub-Saharan Africa [58], [59].

### The African strains have four prevalent fHbp amino acid sequence variants

One hundred percent of the serogroup A isolates, 95% of the X isolates, and 34% of the W-135 isolates had fHbp in the variant 1 group (Figure 2, middle column). The remaining W-135 isolates had fHbp variant 2 (58%) or 3 (8%); one serogroup X isolate had fHbp variant 3.

The distribution of the major fHbp amino acid sequence variants is shown in Figure 2 (right column). All of the group A isolates had fHbp sequence variants ID 4 or 5, which differed from each other by a single amino acid. The most prevalent sequence variants in the serogroup X isolates were ID 74 (67%) and ID 73 (12%); two of the serogroup X isolates in the category “other” had fHbp ID 4, which was prevalent among serogroup A isolates. With only a few exceptions the W-135 isolates were clonal based on having a common clonal complex (CC 11) and PorA VR type (P1.5,2, see Figure S2). This clone could be subdivided into multiple subclones based on genes encoding fHbp ID 9 (variant 1), 22/23 (variant 2, and which differed from each other by one amino acid), or 349 or 111 (variant 3; included in the category “other”, Figure 2). Collectively, four fHbp sequence variants (or related variants, each differing from the respective prevalent variant by 1 amino acid) were present in 81% of the 106 isolates. These were fHbp ID 4/5, 74 or 9 (variant 1 group), or ID 22/23 (variant 2 group). The percent amino acid identities between each of these sequence variants, and between fHbp sequence variants ID 1, 28 and 77, which were used as control vaccines, are summarized in Table 1.

**Table 1:** Amino acid identity between prevalent African fHbp sequence variants, and reference fHbp vaccines.

% AMINO ACID IDENTITY								
Variant Group	ID	1**	4/5*	9*	74*	22/23*	77**	28**
1	**1**	100						
1	**4**	96	100					
1	**9**	94	96	100				
1	**74**	93	92	94	100			
2	**22**	68	68	68	69	100		
2	**77**	71	71	72	73	94	100	
3	**28**	61	59	61	61	88	85	100

Amino acid identity from pair-wise alignments between fHbp sequence variants.

*fHbp sequence variants found among African isolates (ID 4/5 differed from each other by one amino acid as did ID 22/23).

**fHbp sequence variants used as control vaccines.

### Many of the strains from Africa have low fHbp expression

**Figure 3:**
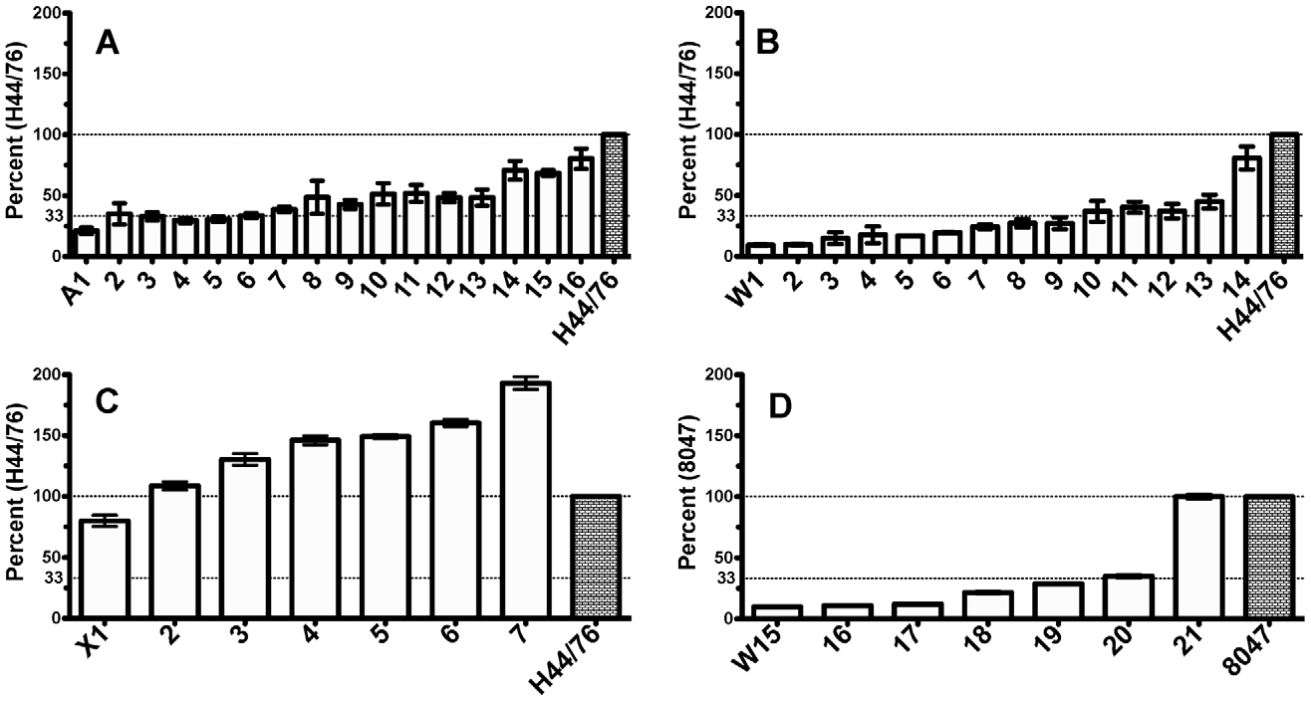
Expression of fHbp by representative African isolates as measured by a quantitative Western blot. Panel A, serogroup A isolates with fHbp ID 4 or 5; Panel B, serogroup W-135 isolates with fHbp ID 9; Panel C, serogroup X isolates with fHbp ID 73 or 74; and Panel D, serogroup W-135 isolates with fHbp ID 22 or 23. Bars in Panels A to C represent mean percentages (ranges) compared with expression of fHbp by the reference group B strain H44/76, which is a relative high expresser of fHbp ID 1 (variant group 1). Bars in Panel D are expression of fHbp compared with expression by reference group B strain 8047, which is a relative high expresser of fHbp ID 77 (variant group 2). Isolates with means below 33% were categorized as low fHbp expressers, and above 100% were defined as high expressers (See text).

We determined fHbp expression levels in bacterial cells from 44 isolates (Figure 3) using a quantitative Western blot. Six of the 16 group A isolates tested (Panel A), and nine of the 14 W-135 isolates tested with fHbp in the variant 1 group (Panel B), had low fHbp expression (defined by ≤33% of fHbp expressed by the group B reference strain H44/76, which is a naturally high expresser of fHbp ID 1 in variant group 1). All but one of seven group W-135 isolates tested with fHbp in the variant group 2 expressed ≤33% of the group B reference strain 8047, which is a naturally high expresser of fHbp ID 77 in variant group 2 (Panel D). In contrast, all but one of the seven group X isolates tested with fHbp in variant 1 group had high fHbp expression (>100% expression compared with H44/76, Panel C). The seventh isolate had ∼80% of the fHbp expressed by H44/76, which was considered intermediate.

### Low fHbp expression is associated with resistance of mutants to anti-fHbp bactericidal activity

**Figure 4:**
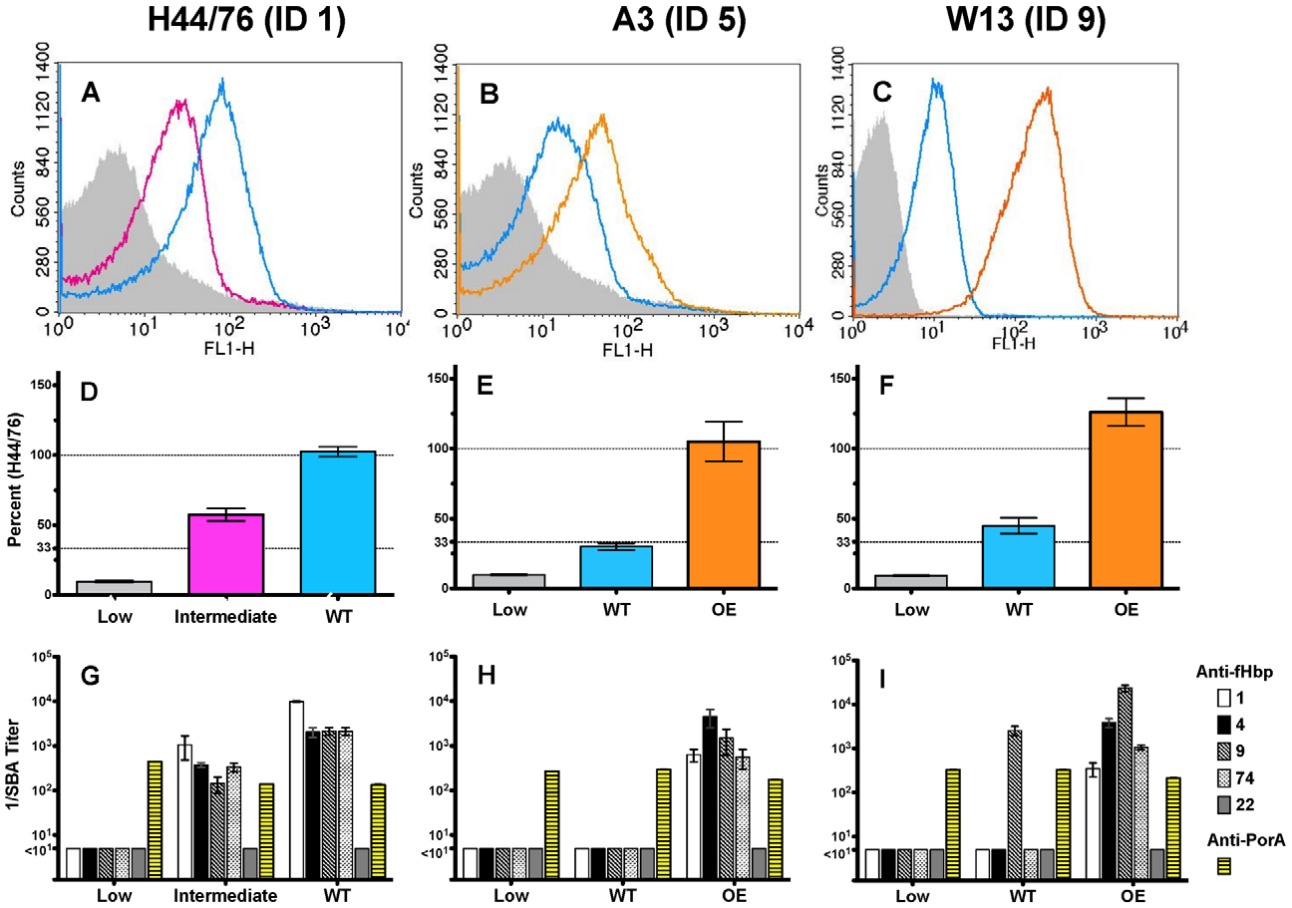
Bactericidal activity of anti-fHbp antisera in relation to fHbp expression by mutant strains. Panels A, B and C, binding of anti-fHbp mAbs JAR 4 and JAR 5 to live bacteria as measured by flow cytometry. Gray, low expression mutant; blue, wildtype expression; magenta, intermediate expression mutant; orange, over-expressed mutant. Panels D, E, and F, fHbp expression measured by quantitative Western blot. Low, ≤33% of expression as compared with H44/76 wildtype strain; “Intermediate”, 34 to 99% expression; and “OE” (over-expressed) mutants of A3 or W13 with 105% and 126%, respectively, of H44/76). Panels G, H and I, bactericidal titers of antisera to recombinant fHbp ID 1 (white bars), 4 (black), 9 (gray, diagonal hatches), or 74 (white, stippled) in variant 1, or ID 22 (gray bars) in variant 2, or anti-PorA, mAbs (yellow bars with horizontal hatches) to P1.7 (H44/76), or P1.9 (A3) or P1.2 (W13). Serogroup B H44/76 is a reference strain with naturally high expression of fHbp ID 1 (variant 1 group). Serogroup A isolate, A3, is a naturally low expresser of fHbp ID 5 from Senegal. Serogroup W-135 isolate, W13, is a naturally low expresser of fHbp ID 9 from the Sudan.

We prepared isogenic mutants with different levels of fHbp expression from three isolates: a serogroup B reference strain, and serogroup A and W-135 African isolates. Figure 4 panels A, B and C illustrate relative expression of fHbp on the surface of live bacteria as measured by flow cytometry. Panels D, E and F show the corresponding fHbp expression measured in solubilized bacterial cells by a quantitative Western blot [50]. For each set of mutants, there was a 5- to 10-fold range between lowest and highest fHbp expression.

The wildtype serogroup B reference isolate had naturally high expression of fHbp ID 1. Sera from mice immunized with each of the recombinant fHbp ID 1, 4, 9, or 74 (variant 1 group) vaccines had high bactericidal titers against this strain (Figure 4, Panel G). As expected, this strain was resistant (titer <1∶10) to the serum from mice immunized with the recombinant fHbp ID 22 vaccine (variant group 2). Against the isogenic mutant of H44/76 with 58% fHbp expression by Western blot as that of the wildtype H44/76 strain, the respective anti-fHbp ID 1, 4, 9, or 74 bactericidal titers were ∼10-fold lower than the corresponding titers measured against the higher fHbp expressing wildtype strain (Panel G). In contrast, none of these antisera was bactericidal against the H44/76 mutant with low fHbp expression (10% of the wildtype isolate). The H44/76 wildtype strain and two fHbp mutants were equally susceptible to bactericidal activity of a positive control mAb against PorA (Panel G, yellow horizontal hatched bars). Similar respective results were observed with the mutants of the serogroup A (A3) and serogroup W-135 isolate (W13). For example, the W-135 wildtype strain with 45% fHbp expression relative to that of the reference strain was susceptible to anti-fHbp bactericidal activity only by the antiserum prepared to the recombinant fHbp vaccine ID 9 that matched that of the target isolate (titer >1∶1000, Panel I). In contrast, the isogenic mutant with increased fHbp expression (126% relative to H44/76) was susceptible to anti-fHbp bactericidal activity by antisera to any of the recombinant fHbp vaccine sequence variants in variant group 1. While the data from the mutants do not permit a precise definition of the level of fHbp expression required for homologous and cross-reactive anti-recombinant fHbp bactericidal activity, collectively the results indicated that fHbp expression below 30% of H44/76 was associated with resistance and, with increasing fHbp expression, the isolates became more susceptible to anti-fHbp cross-reactive bactericidal activity.

### Wildtype strains from Africa are generally susceptible to anti-fHbp antibodies elicited by the recombinant fHbp vaccine that matched the sequence variant of the vaccine but not to antibodies elicited by mismatched recombinant fHbp variants

**Figure 5:**
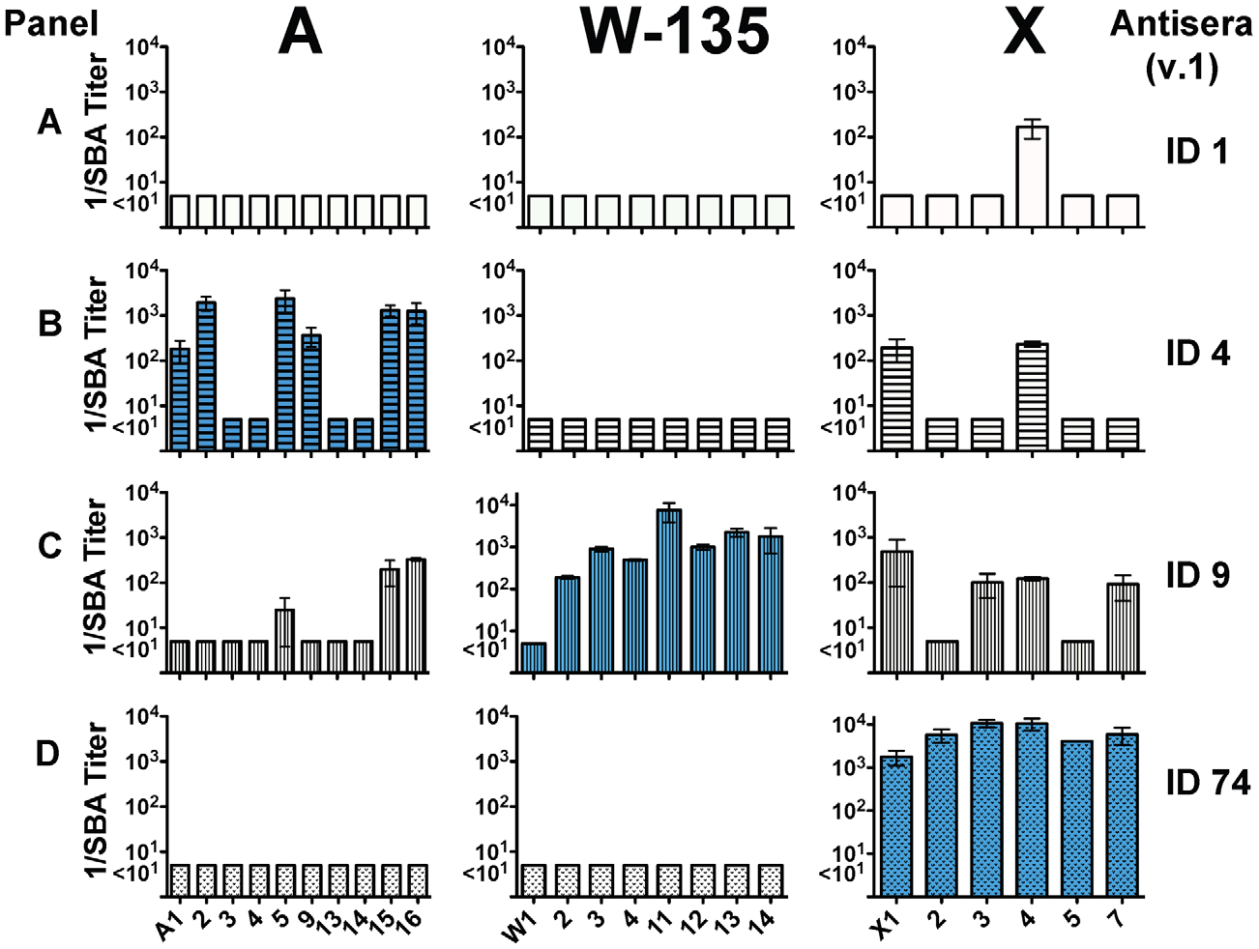
Complement-mediated bactericidal activity of serum pools from mice immunized with recombinant fHbps in variant 1 (v.1) group against strains with fHbp v.1. Panel A, anti-fHbp ID 1; Panel B, anti-fHbp ID 4; Panel C, anti-fHbp ID 9; Panel D, anti-fHbp ID 74. Blue bars highlight responses measured against strains expressing the fHbp sequence variant matching that of the vaccine. Bars represent mean titers ± ranges for individual isolates as measured in at least two independent assays. The anti-fHbp ID 1 antiserum had a titer of 1∶10,240 against a control group B strain with fHbp ID 1 (H44/76, Figure 4, Panel G).

Figure 5 shows the anti-fHbp bactericidal titers of antisera prepared to the different recombinant fHbp sequence variants in variant group 1 when measured against wildtype serogroup A, W-135 and X isolates with fHbp in variant group 1. The isolates were generally susceptible to anti-fHbp antibodies elicited by the recombinant fHbp vaccine that matched the sequence variant of the vaccine (blue bars) but not to antibodies elicited by mismatched recombinant fHbp variants. The lack of cross-reactive bactericidal activity was most notable for the anti-fHbp ID 1 and ID 74 antisera (Panels A and D, respectively). This result was surprising since these vaccines had 92 to 96% amino acid identity with the fHbp expressed by the test strains (Table 1). In Figure 5 the order of the isolates from left to right is with increasing fHbp expression (as shown in Figure 2). While there were trends for increased susceptibility to anti-fHbp bactericidal activity with increasing fHbp expression (for example, the W-135 isolates and the respective anti-fHbp ID 9 titers), the relationship is not linear, being confounded by other variables. The lack of linearity is most evident with the serogroup A isolates where some of the lowest fHbp expressers (i.e., A1 and A2) were killed by the anti-fHbp ID 4 antiserum that matched that of the isolates, while some of the highest expressers (i.e., A13 and A14) were resistant.

**Figure 6:**
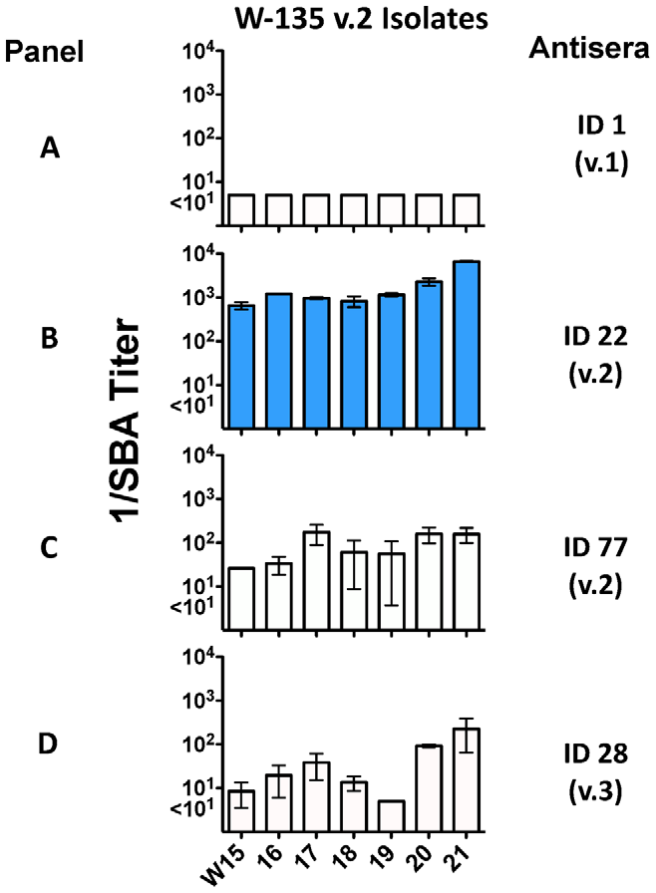
Complement-mediated bactericidal activity of serum pools from mice immunized with recombinant fHbp ID 1 (v.1), ID 22 (v.2), ID 77 (v.2) or ID 28 (v.3) vaccines as measured against serogroup W-135 isolates with fHbp ID 22 or 23. Panel A. anti-fHbp ID 1 (negative control, v.1 antiserum); Panel B, in blue, anti-fHbp ID 22 (matched to fHbp sequence variant of test isolates); Panel C, anti-fHbp ID 77 (mismatched sequence variant in v. 2 group), and Panel D, anti-fHbp ID 28 (mismatched sequence variant in v.3 group). Bars represent mean titers ± ranges for individual isolates as measured in at least two independent assays.

Figure 6 shows the anti-fHbp bactericidal titers of antisera prepared to different recombinant fHbp sequence variants in variant groups 2 or 3 when measured against wildtype serogroup W-135 isolates with fHbp ID22/23 in variant group 2. The anti-fHbp ID 22 antiserum was bactericidal against all of the isolates, even though nearly all of these isolates were low fHbp expressers. Although these isolates also were killed by the control mismatched anti-fHbp ID 77 (variant 2) or fHbp ID 28 (variant 3) antisera (84 to 94 percent amino acid identity with fHbp ID 22/23, Table 1), the respective titers were 10 to 100-fold lower than those of the anti-fHbp ID 22 antiserum (compare anti-ID 22 bactericidal titers in Panel B to those in Panels C and D, Figure 6).

### Antibodies to a native outer membrane vesicle vaccine from a mutant with over-expressed fHbp ID 1 kill African strains resistant to bactericidal activity of antibodies to recombinant fHbp ID 1 vaccine

**Figure 7:**
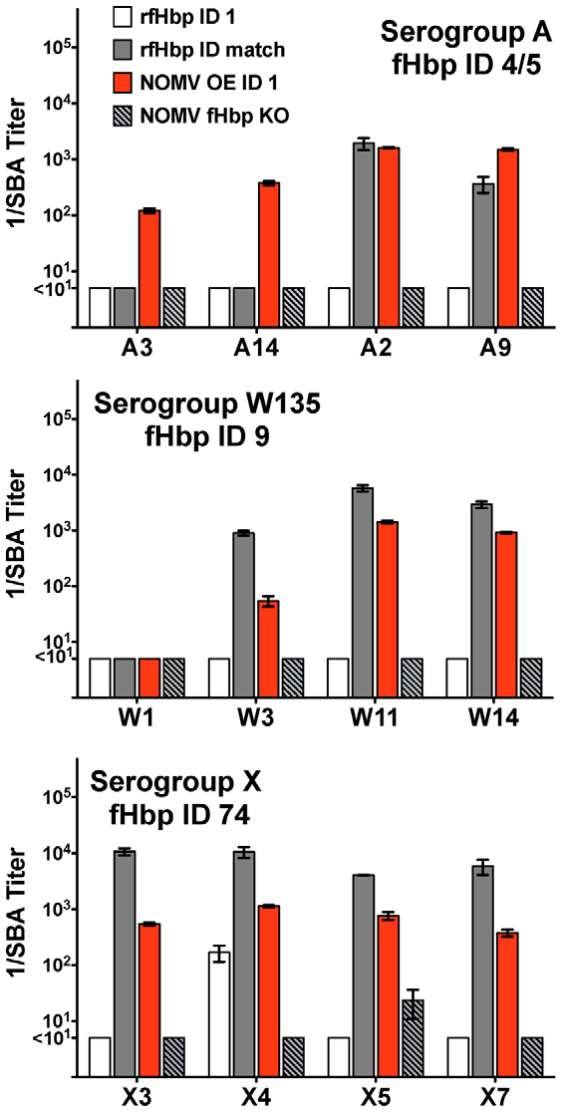
Bactericidal activity of serum pools from mice immunized with recombinant fHbp vaccines, or NOMV vaccines prepared from mutants of group B strain H44/76 with over-expressed or inactivated fHbp ID 1. White bars, recombinant fHbp ID 1; gray bars, recombinant fHbp ID vaccine that matched that of the test isolates (ID 4, serogroup A; ID 9, serogroup W-135; and ID 74, serogroup X); orange bars, NOMV vaccine from a mutant of H44/76 with over-expressed fHbp ID1; gray bars with hatches, NOMV vaccine from a mutant of H44/76 with fHbp knocked-out. Titers are means ± ranges of serum dilutions that gave 50% decreases in CFU/ml after one hr incubation with human complement measured in at least two assays. The dose of recombinant fHbp vaccines was 20 µg, which was administered with Freund's complete for dose 1 and incomplete adjuvant for doses 2 and 3. The dose of the NOMV vaccines was 1.25 µg of total protein adsorbed with 170 µg of aluminum hydroxide.

We measured susceptibility of 12 African isolates to bactericidal activity of an antiserum from mice immunized with a prototype NOMV vaccine prepared from a mutant of group B strain H44/76 with over-expressed fHbp ID 1 (Figure 7). As controls, we tested antisera from mice immunized with an NOMV vaccine from an isogenic fHbp knock-out mutant (NOMV fHbp KO) or a recombinant fHbp ID 1 vaccine. Against the wildtype serogroup B H44/76 strain, which was a high expresser of fHbp ID 1 that matched the recombinant fHbp vaccine, the bactericidal titers of the control antisera to the NOMV vaccine from the fHbp knock-out mutant, or the recombinant fHbp ID 1 vaccine, were both ∼1∶10,240.

Only one of the 12 heterologous African isolates (X4) was susceptible to bactericidal activity of the antiserum to the recombinant fHbp ID 1 vaccine (white bars). In contrast, 11 of the 12 isolates were killed by the antiserum from mice immunized with the NOMV vaccine from the serogroup B mutant with over-expressed fHbp ID 1 (orange bars), which included both serogroup A isolates resistant to bactericidal activity of the antiserum to the recombinant fHbp ID 4 vaccine that matched that of the isolate (gray bars). The one W-135 strain, W1, which was not killed by any of the antisera, was the lowest expresser of fHbp (Figure 3). The remaining three serogroup W-135 tested and all four serogroup X isolates were killed by sera from the mice immunized with the NOMV vaccine from the mutant with over-expressed fHbp ID 1, but the respective bactericidal titers were lower than the corresponding titers elicited by the recombinant fHbp vaccine with a sequence variant that matched that of the strain. With one exception (X5), the bactericidal titers elicited by the control NOMV vaccine from the fHbp knockout mutant were negative (titers <1∶10, gray hatched bars). Although not shown in Figure 7, when we mixed this antiserum with the antiserum to the recombinant fHbp ID 1 vaccine, the serum bactericidal titers remained negative (<1∶10, 11 isolates) or unchanged (titer 1∶50, isolate X5). Thus, there was no evidence of cooperative bactericidal activity between anti-fHbp antibodies elicited by the recombinant fHbp ID 1 vaccine and antibodies elicited to other antigens in the NOMV. These results are in contrast to a previous report of cooperative bactericidal activity observed between human antibodies to recombinant fHbp ID 1 and Neisserial Heparin binding antigen, which individually lacked bactericidal activity [60].

### Bactericidal activity induced by the mutant NOMV vaccine is associated with higher anti-fHbp antibody responses and greater blocking of binding of fH to fHbp than that induced by the recombinant fHbp vaccine

**Figure 8:**
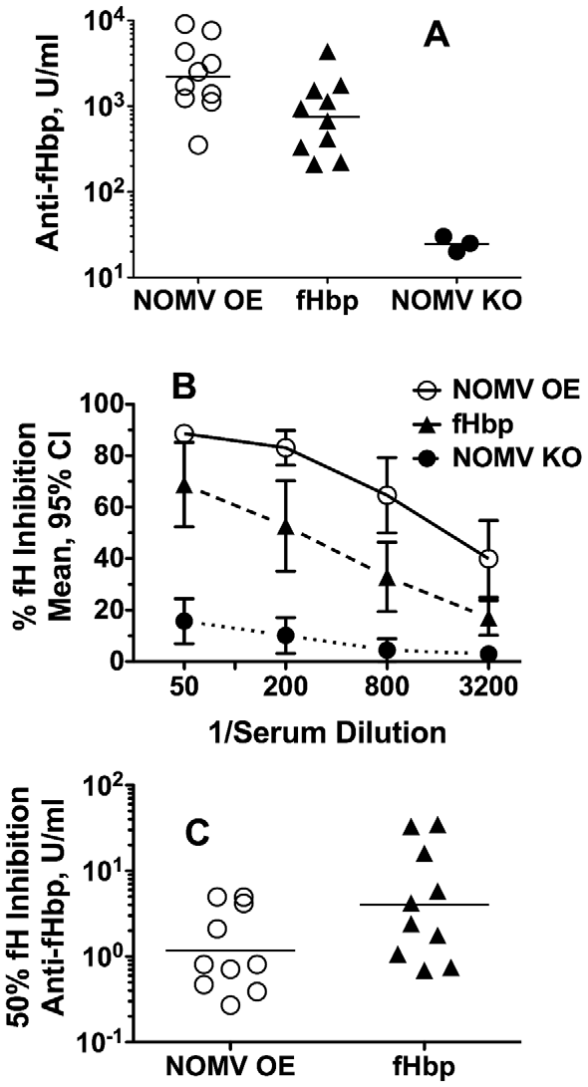
Serum anti-fHbp antibody responses to vaccination as measured by ELISA. Mice were immunized with a recombinant fHbp ID 1 vaccine (filled triangles), or a NOMV vaccine prepared from a mutant of group B strain H44/76 with over-expressed (OE) fHbp ID 1 (open circles), or from a fHbp knock-out (KO) mutant (filled circles). Panel A. Serum anti-fHbp ID 1 antibody concentrations in arbitrary units per ml. NOMV OE vaccine group had a higher geometric mean concentration (horizontal line) than mice immunized with the recombinant fHbp vaccine (p = 0.02). Panel B. Serum anti-fHbp inhibition of binding of fH to fHbp ID 4. At all dilutions tested, the mean percent inhibition of the group given the NOMV vaccine from the mutant with over-expressed fHbp was higher than the recombinant fHbp vaccine group (p<0.05). Panel C. Serum anti-fHbp ID 1 antibody concentrations for 50% inhibition of binding of fH to fHbp ID 4. The geometric mean was lower for the NOMV OE fHbp group than the recombinant fHbp vaccine group (p<0.05).

By ELISA, the mice immunized with the NOMV vaccine from the mutant with over-expressed fHbp ID 1 had higher serum anti-fHbp ID 1 antibody concentrations than mice immunized with the recombinant fHbp ID 1 vaccine (respective geometric means of 2203 and 746 U/ml, P<0.02, Figure 8, Panel A). By ELISA, the sera from the mice immunized with the mutant NOMV vaccine also showed greater inhibition of binding of fH to fHbp ID 4, which was the sequence variant expressed by serogroup A strains (Panel B, P<0.05 at each dilution tested). The increased fH inhibition was not only a result of the higher serum anti-fHbp concentrations in the mutant NOMV vaccine group, but also appeared to be from a different anti-fHbp antibody repertoire, since on average the anti-fHbp antibody concentration required for inhibition of fH in this group was nearly 4-fold lower than in the recombinant fHbp vaccine ID 1 group (respective geometric means of 1.17 vs. 4.04 U/ml, P<0.05, Panel C).

## Discussion

In this study, we investigated 106 serogroup A, X and W-135 isolates from Africa to assess the vaccine-potential of fHbp for prevention of meningococcal epidemics. Our results confirm previous observations that epidemic serogroup A, W-135 and X isolates from sub-Saharan Africa are derived from a limited number of clonal complexes and PorA VR sequence variants [59], [61], [62]. With respect to fHbp, 95 percent of the isolates overall had genes encoding fHbp from variant groups 1 or 2. When we considered fHbp sequence variants that differed by a single amino acid to be a single sequence variant, 81 percent of the had one of four prevalent fHbp amino acid sequence variants: three in variant 1 group and one in variant 2 group.

All of the serogroup A strains obtained over a 45 year period expressed a nearly invariant fHbp ID 4/5 in variant group 1 group. In a recent report, fHbp ID 4/5 (referred to in the article as B16 and B22, respectively) also were prevalent among serogroup A isolates from South Africa, which were from clonal complexes ST-1 and ST-5 [26]. In the present study, two of the three serogroup X isolates from South Africa from the 1970s also had fHbp ID 4 (HF24 and HF78, Table S1) [26]. Further, an identical fHbp variant was prevalent among recent serogroup B isolates from the United Kingdom [63], and serogroup B and Y isolates from South Africa [26]. Collectively, these results illustrate remarkable stability of this fHbp sequence over time. At the opposite extreme with respect to fHbp variability were the serogroup W-135 isolates in our study, which were clonal with respect to sequence type (ST-11) and PorA (P1.5,2), but expressed fHbp sequence variants from variant group 1 (ID 9), 2 (ID 22/23) or 3 (ID 349). Two of these fHbp sequence variants, ID 9 (variant group 1) and ID 22 (variant group 2) also were prevalent among the recent ST-11 W-135 isolates from South Africa (referred to as B45 and A10, respectively) [26]). Therefore, with certain clones, such as ST-11, there is the potential for recombination at the fHbp gene locus, which can result in rapid changes in the fHbp antigenic variant group.

Several previous studies reported broad serum bactericidal activity against serogroup B strains by vaccination with recombinant fHbp antigens from the respective variant group [64] or sub-family [30]. In the present study, however, we found limited cross-reactive serum bactericidal activity in mice immunized with recombinant fHbp vaccines when measured against serogroup A, W-135 or X isolates that did not match the amino acid sequence of vaccine antigen. For the serogroup A and W-135 isolates, low fHbp expression appeared to contribute to resistance to anti-fHbp bactericidal activity. When fHbp is sparsely-exposed on the bacterial surface, the ability of two IgG anti-fHbp antibodies to bind to appropriately space epitopes, engage C1q and activate classical complement pathway bacteriolysis may be limited [45]. Our data from isogenic mutant strains with different levels of fHbp expression directly demonstrated that by increasing fHbp expression, a resistant wildtype serogroup A or serogroup W-135 isolate became more susceptible to anti-fHbp bactericidal activity (Figure 4). Conversely, by decreasing fHbp expression, a susceptible wildtype serogroup B strain became resistant. Among the serogroup X wildtype isolates, however, low expression was not a factor for resistance to bactericidal activity since with one exception these isolates were high fHbp expressers. Low expression also did not appear to explain resistance of two of the wildtype serogroup A isolates resistant to bactericidal activity by antibodies to all of the recombinant fHbp sequence variant vaccines, including the fHbp ID 4 vaccine that matched fHbp in the isolates. These two isolates were killed by control antibodies to the serogroup A capsule and PorA, and by antibodies elicited in mice by the NOMV vaccine from the mutant with increased expression of fHbp ID 1. Further studies are needed to define the basis for resistance of these isolates to bactericidal activity by serum antibodies to the recombinant fHbp vaccines.

The lack of broad cross-reactive bactericidal antibody activity to the African isolates in sera from mice immunized with different recombinant fHbp sequence variants was consistent with recent data from two clinical trials in the UK showing limited breadth of serum bactericidal responses after immunization of infants and toddlers with a multicomponent vaccine containing recombinant fHbp ID 1 [42], [43]. In these trials, broader bactericidal antibody responses were observed when the recombinant proteins were combined with a detergent-treated outer membrane vesicle vaccine, which elicited protective antibodies against PorA and which also had an adjuvant effect that augmented the serum antibody responses to the recombinant proteins.

In previous studies, NOMV vaccines from mutants with over-expressed fHbp elicited broad serum bactericidal antibody responses in mice against genetically diverse serogroup B strains [35], [36], [51], [54]. Broad serum bactericidal responses against serogroup B strains also were recently described in an infant non-human primate model [33]. In the present study, mice immunized with an NOMV vaccine from a group B mutant with over-expressed fHbp ID 1 developed serum bactericidal activity against all but one of the isolates tested from Africa. In contrast, all but one of the isolates were resistant to the antiserum to the recombinant fHbp ID 1 vaccine. For the NOMV vaccines, each mouse received 1.25 µg of total protein, which was adsorbed with 170 µg of aluminum hydroxide. For the recombinant protein vaccines, the mice were immunized with 20 µg of recombinant protein mixed with Freund's complete adjuvant for the first dose and Freund's incomplete adjuvant for doses 2 and 3. If anything, the broad serum bactericidal titers elicited by the lower dose of NOMV vaccine given with the aluminum adjuvant, which is suitable for use in humans, underscores the greater vaccine potential of this approach to elicit broad protective immunity. At least three factors appeared to contribute to the enhanced serum bactericidal antibody responses to the NOMV vaccine with increased fHbp expression. First, was over-expression of fHbp, which in a recent study was shown to be required for broad serum anti-fHbp bactericidal responses [34]. Second, was the presence of natural adjuvants in the NOMV such as lipooligosaccharide or PorB [65]. Third, was the possible effect of fHbp antigen presentation in the NOMV on anti-fHbp antibody repertoire as evidenced by greater ability of the anti-fHbp antibodies to inhibit of binding of fH to fHbp than anti-fHbp antibodies elicited by the recombinant fHbp ID 1 vaccine (Figure 8). Greater inhibition of binding of fH to the surface of *N. meningitidis* also would be expected to decrease down-regulation of complement activation by fH, and enhance susceptibility of the organism to bactericidal activity [37], [38].

In summary, the present data from studies in mice immunized with a prototype NOMV vaccine with increased fHbp expression suggest that this vaccine approach could supplement coverage conferred by the serogroup A polysaccharide conjugate vaccine recently introduced in Africa, and extend coverage against strains with other serogroups. An important question, however, is whether a mutant NOMV vaccine that requires several doses for protection is likely to be practical in a resource poor region such as Africa as compared with conjugate vaccines against serogoups X and W-135. In older children and adults, a single dose of a meningococcal polysaccharide conjugate vaccine can elicit protective serum antibodies. Protection, however, elicited by conjugate vaccines is serogroup specific whereas NOMV vaccines with over-expressed fHbp elicit protective antibodies against strains with different serogroup capsules. Also, in infants immunized with a conjugate vaccine, more than one primary dose usually is necessary for protection [66], and protective serum antibodies last less than a year or two [67], [68]. Thus, periodic boosting with a conjugate vaccine is required to maintain immunity [68], [69]. Defining an optimal NOMV vaccine schedule will require studies in humans. That multiple NOMV doses may be needed to elicit protective antibodies, however, is not necessarily different from the requirements for eliciting and maintaining protection by meningococcal conjugate vaccines in infants and children.

### Limitations of the study

There are several important limitations to the present study. First, we investigated only 106 case isolates. Although these isolates were from 17 countries, 30 percent were from Burkina Faso. Africa is a large and diverse continent with a complex ecology affecting meningococcal transmission and disease. Development of a mutant NOMV fHbp-based vaccine for sub-Saharan Africa will require ongoing surveillance of meningococcal strains to assure that the vaccine antigens match those of the prevalent strains. Second, for investigation of anti-fHbp antibody activity elicited by the recombinant fHbp vaccines, we prepared hyperimmune antiserum pools in mice immunized with the vaccines given with Freunds complete and incomplete adjuvant. This adjuvant is unsuitable for humans and the high anti-fHbp titers in the hyperimmune mouse serum pools are unlikely to be achieved in humans. The poor cross-reactive bactericidal activity of these mouse antisera is, therefore, likely to be even lower in humans immunized with recombinant vaccines given with aluminum adjuvants. The broad serum bactericidal antibody responses to the mutant fHbp NOMV vaccine, however, were in mice given the vaccine with aluminum hydroxide.

A third limitation was that while the data from the isogenic mutants with different levels of fHbp showed a clear relationship between fHbp expression levels and susceptibility to anti-fHbp bactericidal activity, the relatively small number of wild type strains tested, and the presence of potential confounders such as capsular serogroup [70], LOS [71], [72] or alternative fH binding ligands [73], did not provide sufficient statistical power for a formal analysis of the relationship between fHbp expression and anti-fHbp bactericidal activity.

A fourth limitation of the present study is that we used a prototype NOMV vaccine from a mutant group B strain with over-expressed fHbp ID 1. We chose this vaccine since it had worked well in previous studies of group B strains, and an NOMV vaccine from a similar mutant African strain was not yet available. Neither the PorA VR type of the group B vaccine strain nor the fHbp sequence variant was present among the African isolates. We would anticipate that even higher serum bactericidal antibody responses would be elicited by NOMV vaccines prepared from mutant African isolates where the antigens in the vaccine would be matches to the Africa strains. Finally, although the serum bactericidal titers of the control mice immunized with the NOMV from the fHbp knockout were negative, we had insufficient sera from mice immunized with the NOMV vaccine from the mutant with over-expressed fHbp to prove that the bactericidal antibodies were directed against fHbp. In several previous studies however, we demonstrated that bactericidal activity against serogroup B strains was greatly or completely diminished after depletion of anti-fHbp antibodies by solid phase adsorption [34], [36], [51].

## Supporting Information

Figure S1 **Characterization of NOMV vaccines.** Panel A. Expression of fHbp as measured by Western-blot with anti-fHbp mAb JAR 3. Lane 1, rfHbp ID 1, purified His-tagged protein expressed in *E. coli*; Lane 2, NOMV from H44/76 fHbp KO mutant; Lane 3, NOMV from mutant with over-expressed fHbp ID 1; Lane 4, NOMV from wildtype H44/76 strain. Panel B. SDS-PAGE and Coomassie blue stain of NOMV vaccines. M, Molecular mass markers; Lane 2, fHbp knock-out mutant; Lane 3, Mutant with over-expressed fHbp. The NOMV from the fHbp over-expressed mutant showed higher amounts of a band resolving at ∼30kD. In parallel experiments the band resolving in this portion of the gel contained fHbp and OpcA. Panel C. Silver stained SDS-PAGE of LOS in NOMV vaccines from fHbp KO mutant (Lane 2) or fHbp over-expressed mutant (Lane 3). Amounts of vesicles loaded in each lane were standardized based on total protein content.
(TIF)

Figure S2 **Distribution of PorA variable region (VR) types among African isolates.** PorA VR type designations were made as described at http://pubmlst.org/neisseria/. Among the serogroup A isolates, P1.20,9 was present overall in 55%, and in 89% of 18 serogroup A isolates obtained since 1990. Among the serogroup W- 135 isolates, P1.5,2 and related types such as 5-1,2-2 predominated (98%), and among the serogroup X isolates, P1.19,26 and a related type P1.19,26-4 accounted for 68%. These results are consistent with previous studies of strains from sub-Saharan Africa [58], [59].
(TIF)

Table S1 **Characteristic of meningococcal strain panel.** *Designation used in labels of figures, table and text of paper. ^†^UA, undesignated. Note ST 3687 and ST 5403 differed by only a single nucleotide change in one of the seven loci (*fumC)* and, thus, may represent a new CC. Novartis variant groups as described by Masignani et al [27]; Pfizer subfamilies as described by Fletcher et al [28]; Modular groups as described by Beernink & Granoff [53] and Pajon et al [50]. These amino acid sub-family and variant group designations and the fHbp ID can be found at http://pubmlst.org/neisseria/fHbp/.
(DOC)
