# Supplementary figures and images for: Meningococcal Factor H Binding Proteins in Epidemic Strains from Africa: Implications for Vaccine Development
Source: PLoS Negl Trop Dis. 2011 Sep 6;5(9):e1302. doi: 10.1371/journal.pntd.0001302 (PMC3167780; doi:10.1371/journal.pntd.0001302)

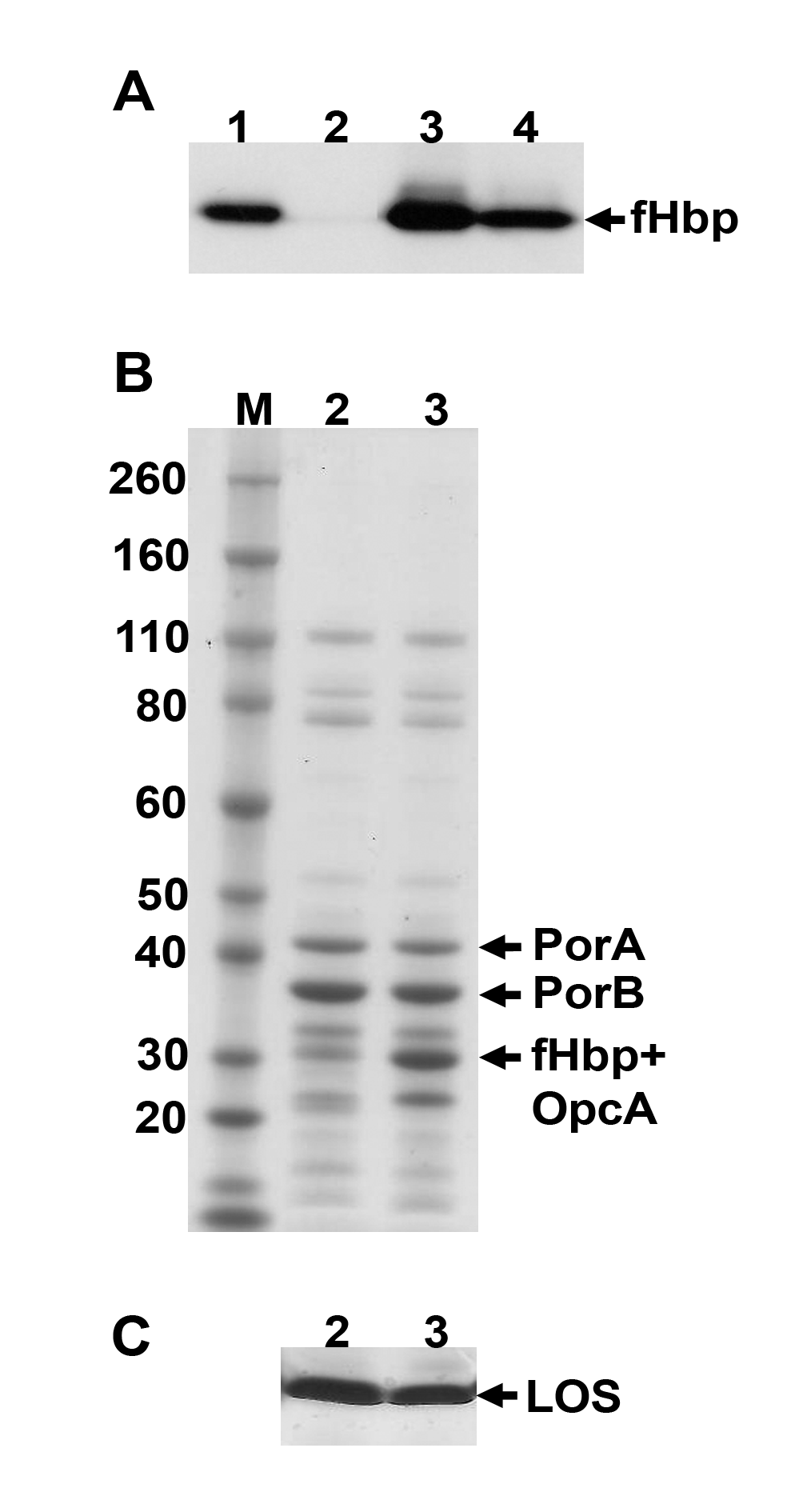

Supplement: Figure S1 — Characterization of NOMV vaccines. Panel A. Expression of fHbp as measured by Western-blot with anti-fHbp mAb JAR 3. Lane 1, rfHbp ID 1, purified His-tagged protein expressed in E. coli; Lane 2, NOMV from H44/76 fHbp KO mutant; Lane 3, NOMV from mutant with over-expressed fHbp ID 1; Lane 4, NOMV from wildtype H44/76 strain. Panel B. SDS-PAGE and Coomassie blue stain of NOMV vaccines. M, Molecular mass markers; Lane 2, fHbp knock-out mutant; Lane 3, Mutant with over-expressed fHbp. The NOMV from the fHbp over-expressed mutant showed higher amounts of a band resolving at ∼30kD. In parallel experiments the band resolving in this portion of the gel contained fHbp and OpcA. Panel C. Silver stained SDS-PAGE of LOS in NOMV vaccines from fHbp KO mutant (Lane 2) or fHbp over-expressed mutant (Lane 3). Amounts of vesicles loaded in each lane were standardized based on total protein content. (TIF) [file pntd.0001302.s001.tif]

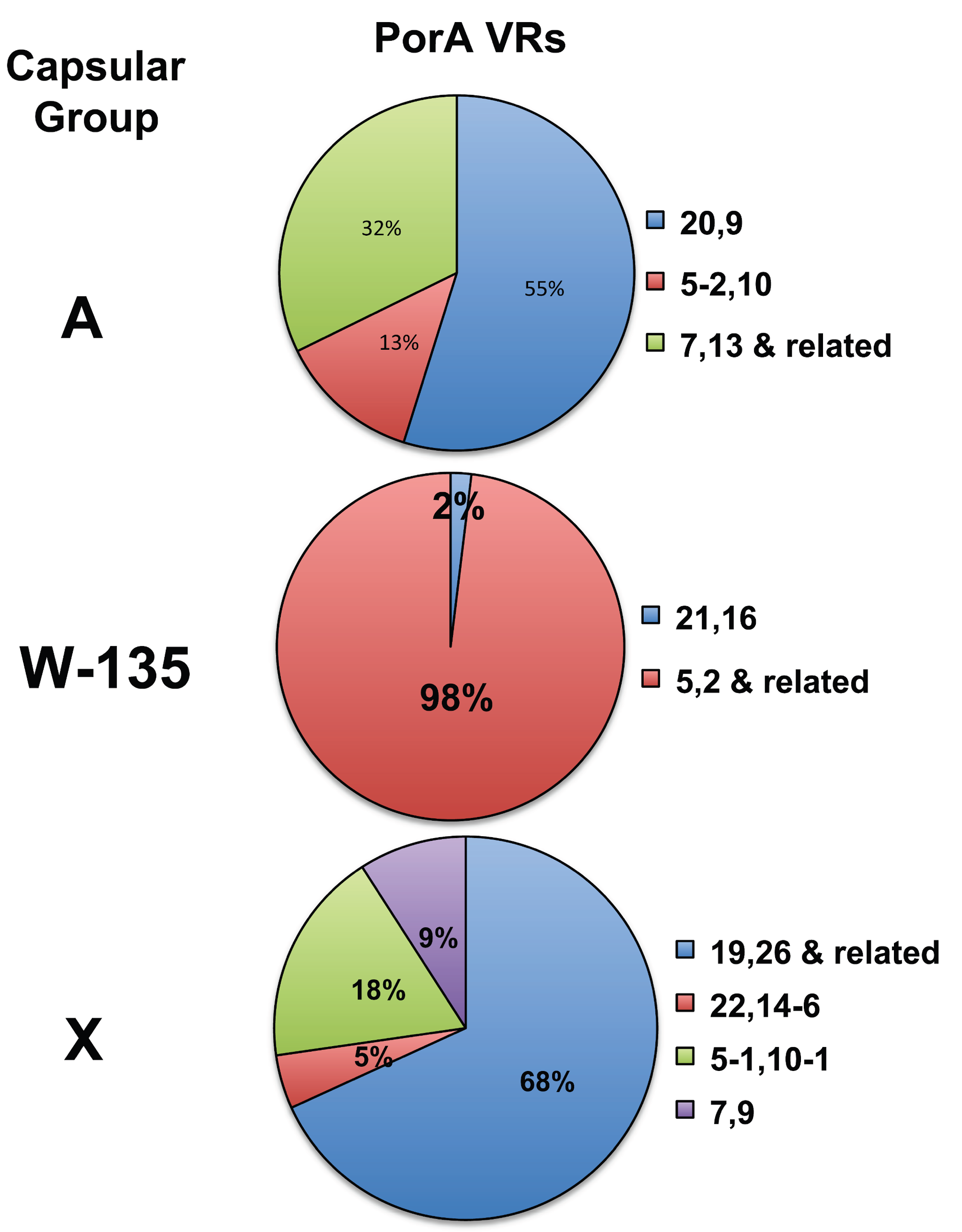

Supplement: Figure S2 — Distribution of PorA variable region (VR) types among African isolates. PorA VR type designations were made as described at http://pubmlst.org/neisseria/. Among the serogroup A isolates, P1.20,9 was present overall in 55%, and in 89% of 18 serogroup A isolates obtained since 1990. Among the serogroup W- 135 isolates, P1.5,2 and related types such as 5-1,2-2 predominated (98%), and among the serogroup X isolates, P1.19,26 and a related type P1.19,26-4 accounted for 68%. These results are consistent with previous studies of strains from sub-Saharan Africa [58], [59]. (TIF) [file pntd.0001302.s002.tif]
