# Supplementary material for: Meningococcal Factor H Binding Proteins in Epidemic Strains from Africa: Implications for Vaccine Development
Source: PLoS Negl Trop Dis. 2011 Sep 6;5(9):e1302. doi: 10.1371/journal.pntd.0001302 (PMC3167780; doi:10.1371/journal.pntd.0001302)
Supplement: Table S1 — Characteristic of meningococcal strain panel. *Designation used in labels of figures, table and text of paper. †UA, undesignated. Note ST 3687 and ST 5403 differed by only a single nucleotide change in one of the seven loci (fumC) and, thus, may represent a new CC. Novartis variant groups as described by Masignani et al [27]; Pfizer subfamilies as described by Fletcher et al [28]; Modular groups as described by Beernink & Granoff [53] and Pajon et al [50]. These amino acid sub-family and variant group designations and the fHbp ID can be found at http://pubmlst.org/neisseria/fHbp/. (DOC) [file pntd.0001302.s003.doc]

**Table S1. Characteristic of meningococcal strain panel**

|  | **Strain Name** | **Secondary Strain Designation*** | **Capsular Group** | **Country** | **Year** | **fHbp Protein ID** | **Novartis variant family** | **Pfizer sub-family** | **Modular Group** | **Clonal Complex [ST]** | ***porA VRs*** | **Reference** |
| --- | --- | --- | --- | --- | --- | --- | --- | --- | --- | --- | --- | --- |
| 1 | Z1269 |  | A | Burkina Faso | 1963 | 5 | 1 | B | I | 4 [4] | P1.7,13-1 | [74] |
| 2 | Z1318 | A1 | A | Burkina Faso | 1966 | 5 | 1 | B | I | 4 [4] | P1.7-5,13-1 | [74] |
| 3 | LNP20868 | A13 | A | Burkina Faso | 2003 | 5 | 1 | B | I | 5 [2859] | P1.20,9 | [49] |
| 4 | LNP20790 |  | A | Burkina Faso | 2003 | 5 | 1 | B | I | 5 [2859] | P1.20,9 | [49] |
| 5 | BuFa6/07 | A9 | A | Burkina Faso | 2007 | 5 | 1 | B | I | 5 [2859] | P1.20,9 | [49] |
| 6 | BuFa20030378 |  | A | Burkina Faso | 2007 | 5 | 1 | B | I | 5 [6035] | P1.20,9 | this work |
| 7 | Z1362 |  | A | Cameroon | 1966 | 5 | 1 | B | I | 4 [4] | P1.7,13 | [74] |
| 8 | F6124 |  | A | Chad | 1988 | 5 | 1 | B | I | 5 [5] | P1.20,9 | [75] |
| 9 | Chad5/07 | A5 | A | Chad | 2007 | 5 | 1 | B | I | 5 [7] | P1.20,9 | this work |
| 10 | C50/01 | A12 | A | Congo | 2001 | 5 | 1 | B | I | 5 [7] | P1.20,9 | this work |
| 11 | Z5010 |  | A | Djibouti | 1966 | 4 | 1 | B | I | 1 [1] | P1.5-2,10 | [74] |
| 12 | E23/03 | A14 | A | Ethiopia | 2003 | 5 | 1 | B | I | 5 [7] | P1.20,9 | [49] |
| 13 | E2/88 | A8 | A | Ethiopia | 2003 | 5 | 1 | B | I | 5 [7] | P1.20,9 | [49] |
| 14 | Z2491 | A16 | A | Gambia | 1983 | 5 | 1 | B | I | 4 [4] | P1.7,13-1 | [76] |
| 15 | C2 |  | A | Gambia | 1988 | 4 | 1 | B | I | 4 [4] | P1.7,13-1 | this work |
| 16 | Z1213 |  | A | Ghana | 1973 | 5 | 1 | B | I | 4 [4] | P1.7,13-1 | [49] |
| 17 | Z4421 |  | A | Mali | 1990 | 5 | 1 | B | I | 4 [4] | P1.7,13 | [74] |
| 18 | Z4186 |  | A | Mali | 1990 | 5 | 1 | B | I | 4 [4] | P1.7,13-1 | [75] |
| 19 | M1/07 |  | A | Mali | 2007 | 5 | 1 | B | I | 5 [7] | P1.20,9 | this work |
| 20 | Z5005 |  | A | Morocco | 1967 | 4 | 1 | B | I | 1 [1] | P1.5-2,10 | [74] |
| 21 | Z1275 | A15 | A | Niger | 1963 | 4 | 1 | B | I | 1 [1] | P1.5-2,10 | [74] |
| 22 | Z1278 | A10 | A | Niger | 1963 | 5 | 1 | B | I | 4 [4] | P1.7,13 | [49] |
| 23 | Niger1/95 | A6 | A | Niger | 1995 | 5 | 1 | B | I | 5 [5] | P1.20,9 | [49] |
| 24 | Niger12/06 | A2 | A | Niger | 2006 | 5 | 1 | B | I | 5 [7] | P1.20,9 | [49] |
| 25 | Nigeria4/03 | A11 | A | Nigeria | 2003 | 5 | 1 | B | I | 5 [7] | P1.20,9 | this work |
| 26 | Niga3/07 | A7 | A | Nigeria | 2007 | 5 | 1 | B | I | 5 [7] | P1.20,9 | [49] |
| 27 | SB7 |  | A | S. Africa | 1989 | 4 | 1 | B | I | 1 [8244] | P1.5-2,10 | this work |
| 28 | Senegal1/99 | A3 | A | Senegal | 1999 | 5 | 1 | B | I | 5 [5] | P1.20,9 | [49] |
| 29 | Z3667 |  | A | Sudan | 1985 | 5 | 1 | B | I | 4 [4] | P1.7,13-1 | [74] |
| 30 | S1/99 |  | A | Sudan | 1999 | 5 | 1 | B | I | 5 [7] | P1.20,9 | this work |
| 31 | Sudan11/07 | A4 | A | Sudan | 2007 | 5 | 1 | B | I | 5 [7] | P1.20,9 | this work |
| 32 | BF 7/01 | W8 | W-135 | Burkina Faso | 2001 | 9 | 1 | B | I | 11 [11] | P1.5,2 | this work |
| 33 | BF 10/01 | W5 | W-135 | Burkina Faso | 2001 | 9 | 1 | B | I | 11 [11] | P1.5,2 | this work |
| 34 | BF 14/01 | W4 | W-135 | Burkina Faso | 2001 | 9 | 1 | B | I | 11 [11] | P1.5,2 | this work |
| 35 | BF 17/01 | W1 | W-135 | Burkina Faso | 2001 | 9 | 1 | B | I | 11 [11] | P1.5,2 | this work |
| 36 | BuFa16/01 | W2 | W-135 | Burkina Faso | 2001 | 9 | 1 | B | I | 11 [11] | P1.5,2 | [49] |
| 37 | BF 10/02 |  | W-135 | Burkina Faso | 2002 | 23 | 2 | A | III | 11 [11] | P1.5,2 | this work |
| 38 | BF 11/02 |  | W-135 | Burkina Faso | 2002 | 23 | 2 | A | III | 11 [11] | P1.5,2 | this work |
| 39 | BF 12/02 |  | W-135 | Burkina Faso | 2002 | 23 | 2 | A | III | 11 [11] | P1.5,2 | this work |
| 40 | BF 13/02 |  | W-135 | Burkina Faso | 2002 | 23 | 2 | A | III | 11 [11] | P1.5,2 | this work |
| 41 | BF 14/02 |  | W-135 | Burkina Faso | 2002 | 23 | 2 | A | III | 11 [11] | P1.5,2 | this work |
| 42 | BF 15/02 |  | W-135 | Burkina Faso | 2002 | 23 | 2 | A | III | 11 [11] | P1.5,2 | this work |
| 43 | BF 21/02 |  | W-135 | Burkina Faso | 2002 | 23 | 2 | A | III | 11 [11] | P1.5,2 | this work |
| 44 | BF 23/02 |  | W-135 | Burkina Faso | 2002 | 23 | 2 | A | III | 11 [11] | P1.5,2 | this work |
| 45 | BF 24/02 |  | W-135 | Burkina Faso | 2002 | 23 | 2 | A | III | 11 [11] | P1.5,2 | this work |
| 46 | BF 25/02 |  | W-135 | Burkina Faso | 2002 | 23 | 2 | A | III | 11 [1966] | P1.5,2 | this work |
| 47 | BuFa6/02 | W17 | W-135 | Burkina Faso | 2002 | 23 | 2 | A | III | 11 [11] | P1.5,2 | [49] |
| 48 | BuFa16/02 | W16 | W-135 | Burkina Faso | 2002 | 23 | 2 | A | III | 11 [11] | P1.5,2 | [49] |
| 49 | M9261 |  | W-135 | Burkina Faso | 2002 | 23 | 2 | A | III | 11 [11] | P1.5,2 | [49] |
| 50 | M9262 |  | W-135 | Burkina Faso | 2002 | 23 | 2 | A | III | 11 [11] | P1.5,2 | [49] |
| 51 | BuFa1/03 |  | W-135 | Burkina Faso | 2003 | 23 | 2 | A | III | 11 [11] | P1.5,2 | [49] |
| 52 | BuFa2-03 | W15 | W-135 | Burkina Faso | 2003 | 23 | 2 | A | III | 11 [11] | P1.5,2 | [49] |
| 53 | BF2/06 | W12 | W-135 | Burkina Faso | 2006 | 9 | 1 | B | I | 11 [5779] | P1.5,2 | this work |
| 54 | Chad2/03 | W7 | W-135 | Chad | 2003 | 9 | 1 | B | I | 11 [11] | P1.5,2 | this work |
| 55 | Chad1/06 | W10 | W-135 | Chad | 2006 | 9 | 1 | B | I | 11 [11] | P1.5,2 | this work |
| 56 | C6/01 |  | W-135 | Congo | 2001 | 350 | 1 | B | I | 175 [175] | P1.5-1,2-2 | this work |
| 57 | 1724 |  | W-135 | Gambia | 1995 | 151 | 2 | A | VI | 11 [11] | P1.5,2 | this work |
| 58 | 2767 |  | W-135 | Gambia | 1995 | 151 | 2 | A | VI | 11 [11] | P1.5,2 | this work |
| 59 | 2040 |  | W-135 | Gambia | 1995 | 111 | 3 | A | II | 11 [11] | P1.5,2 | this work |
| 60 | 2307 |  | W-135 | Gambia | 1995 | 348 | 1 | B | I | 11 [11] | P1.5,2 | this work |
| 61 | 385 |  | W-135 | Gambia | 1995 | 151 | 2 | A | VI | 11 [11] | P1.5,2 | this work |
| 62 | G68 |  | W-135 | Gambia | 1980s | 21 | 2 | A | III | 174 [174] | P1.21,16 | this work |
| 63 | G769 |  | W-135 | Gambia | 1980s | 22 | 2 | A | III | 175 [175] | P1.5-1,2-2 | this work |
| 64 | Ghana 6/04 | W19 | W-135 | Ghana | 2004 | 23 | 2 | A | III | 11 [11] | P1.5,2 | this work |
| 65 | Ghana 7/04 | W21 | W-135 | Ghana | 2004 | 23 | 2 | A | III | 11 [11] | P1.5,2 | this work |
| 66 | MK82/94 |  | W-135 | Mali | 1994 | 151 | 2 | A | VI | 11 [11] | P1.5,2 | this work |
| 67 | MK87/94 |  | W-135 | Mali | 1994 | 22 | 2 | A | III | 11 [11] | P1.5,2 | this work |
| 68 | Mali 1/06 | W20 | W-135 | Mali | 2006 | 23 | 2 | A | III | 11 [11] | P1.5,2 | this work |
| 69 | Mali 2/06 |  | W-135 | Mali | 2006 | 23 | 2 | A | III | 11 [11] | P1.5,2 | this work |
| 70 | Mali 3/06 | W9 | W-135 | Mali | 2006 | 9 | 1 | B | I | 11 [11] | P1.5,2 | this work |
| 71 | Mali 4/06 | W11 | W-135 | Mali | 2006 | 9 | 1 | B | I | 11 [11] | P1.5,2 | this work |
| 72 | Mali 5/06 |  | W-135 | Mali | 2006 | 23 | 2 | A | III | 11 [11] | P1.5,2 | this work |
| 73 | Mali29-07 | W14 | W-135 | Mali | 2007 | 9 | 1 | B | I | 11 [11] | P1.5,2 | [49] |
| 74 | Nigeria 1/03 | W18 | W-135 | Nigeria | 2003 | 22 | 2 | A | III | 175 [2881] | P1.5-1,2-36 | this work |
| 75 | Nigeria 2/03 |  | W-135 | Nigeria | 2003 | 22 | 2 | A | III | 175 [2881] | P1.5-1,2-36 | this work |
| 76 | Nigeria1/04 |  | W-135 | Nigeria | 2004 | 22 | 2 | A | III | 175 [2881] | P1.5-1,2-36 | this work |
| 77 | Sudan 3/06 |  | W-135 | Sudan | 2006 | 9 | 1 | B | I | 11 [11] | P1.5,2 | this work |
| 78 | Sudan 4/06 | W6 | W-135 | Sudan | 2006 | 9 | 1 | B | I | 11 [11] | P1.5,2 | this work |
| 79 | Su1/06 | W13 | W-135 | Sudan | 2006 | 9 | 1 | B | I | 11 [11] | P1.5,2 | [49] |
| 80 | Uganda 11/06 |  | W-135 | Uganda | 2006 | 349 | 3 | A | II | 11 [11] | P1.5,2 | this work |
| 81 | Uganda12/06 |  | W-135 | Uganda | 2006 | 349 | 3 | A | II | 11 [11] | P1.5,2 | this work |
| 82 | Uganda 15/06 |  | W-135 | Uganda | 2006 | 349 | 3 | A | II | 11 [11] | P1.5,2 | this work |
| 83 | Uganda 18/06 | W3 | W-135 | Uganda | 2006 | 9 | 1 | B | I | 11 [11] | P1.5,2 | this work |
| 84 | Uganda 19/06 |  | W-135 | Uganda | 2006 | 9 | 1 | B | I | 11 [11] | P1.5,2 | this work |
| 85 | BF 5/97 |  | X | Burkina Faso | 1997 | 73 | 1 | B | I | 181 [751] | P1.5-1,10-1 | this work |
| 86 | BuFa2-97 |  | X | Burkina Faso | 1997 | 73 | 1 | B | I | 181 [751] | P1.5-1,10-1 | [49] |
| 87 | BF 12/03 | X1 | X | Burkina Faso | 2003 | 73 | 1 | B | I | 181 [751] | P1.5-1,10-1 | this work |
| 88 | BuFa7/07 | X2 | X | Burkina Faso | 2007 | 74 | 1 | B | I | 181 [181] | P1.5-1,10-1 | [49] |
| 89 | Kenya1/06 | X7 | X | Kenya | 2006 | 74 | 1 | B | I | UA^†^ [5403] | P1.19,26 | this work |
| 90 | HF24 |  | X | S. Africa | 1970s | 4 | 1 | B | I | UA^†^ [3687] | P1.7,9 | this work |
| 91 | HF78 |  | X | S. Africa | 1970s | 4 | 1 | B | I | UA^†^ [3687] | P1.7,9 | this work |
| 92 | HF94 |  | X | S. Africa | 1970s | 351 | 3 | A | II | UA^†^ [3687] | P1.22,14-6 | this work |
| 93 | Uganda 3/06 |  | X | Uganda | 2006 | 74 | 1 | B | I | UA^†^ [5403] | P1.19,26 | this work |
| 94 | Uganda 5/06 |  | X | Uganda | 2006 | 74 | 1 | B | I | UA^†^ [5403] | P1.19,26 | this work |
| 95 | Uganda 9/06 |  | X | Uganda | 2006 | 74 | 1 | B | I | UA^†^ [5403] | P1.19,26 | this work |
| 96 | Uganda14/06 | X4 | X | Uganda | 2006 | 74 | 1 | B | I | UA^†^ [5403] | P1.19,26-4 | this work |
| 97 | Uganda 21/06 |  | X | Uganda | 2006 | 74 | 1 | B | I | UA^†^ [5403] | P1.19,26 | this work |
| 98 | Uganda 22/06 |  | X | Uganda | 2006 | 74 | 1 | B | I | UA^†^ [5403] | P1.19,26 | this work |
| 99 | Uganda 23/06 | X6 | X | Uganda | 2006 | 74 | 1 | B | I | UA^†^ [5403] | P1.19,26 | this work |
| 100 | Ug10-06 |  | X | Uganda | 2006 | 74 | 1 | B | I | UA^†^ [5403] | P1.19,26 | [49] |
| 101 | Uganda 5/07 | X5 | X | Uganda | 2007 | 74 | 1 | B | I | UA^†^ [5403] | P1.19,26 | this work |
| 102 | Uganda 6/07 |  | X | Uganda | 2007 | 74 | 1 | B | I | UA^†^ [5403] | P1.19,26 | this work |
| 103 | Uganda 7/07 |  | X | Uganda | 2007 | 74 | 1 | B | I | UA^†^ [5403] | P1.19,26 | this work |
| 104 | Uganda10/07 | X3 | X | Uganda | 2007 | 74 | 1 | B | I | UA^†^ [5403] | P1.19,26 | this work |
| 105 | Uganda 11/07 |  | X | Uganda | 2007 | 74 | 1 | B | I | UA^†^ [5403] | P1.19,26 | this work |
| 106 | Ug13/07 |  | X | Uganda | 2007 | 74 | 1 | B | I | UA^†^ [5403] | P1.19,26 | [49] |
